# The Arrival of Highly Pathogenic Avian Influenza Viruses in North America, Ensuing Epizootics in Poultry and Dairy Farms and Difficulties in Scientific Naming

**DOI:** 10.1111/1751-7915.70062

**Published:** 2024-12-06

**Authors:** Harald Brüssow

**Affiliations:** ^1^ Department of Biosystems Laboratory of Gene Technology, KU Leuven Leuven Belgium

**Keywords:** Avian influenza viruses, dairy farms, epizootics, high pathogenicity avian influenza virus, poultry outbreak, spillover infections

## Abstract

The highly pathogenic avian influenza virus (HPAIV) H5N1, first isolated in 1996 in China, spread rapidly across Eurasia and caused major epizootics in wild and domesticated birds, as well as spillover infections in humans characterised by high mortality. Avian influenza viruses are therefore candidate viruses for a human pandemic. Surprisingly, HPAIV was not isolated in North America until 2014. With the help of intensive biological sampling and viral genome sequencing, the intrusion of HPAIV into North America could be retraced to two separate events. First, migratory birds carried HPAIV from East Siberia via Beringia and dispersed the virus along the Pacific flyway. After reassortment with genes of local low pathogenic avian influenza viruses, HPAIV H5 caused 2015 a major epizootic on poultry farms in the US Mid‐West. After costly containment, HPAIV dropped below the detection limit. In 2021, Eurasian HPAIV H5 viruses arrived a second time in North America, carried by migratory birds to Canada via the Atlantic flyway, using Iceland as a stop. The H5 virus then spread with water birds along the East Coast of the United States and dispersed across the United States. In contrast to the 2015 poultry outbreak, spillover infections into diverse species of mammals were now observed. The events culminated in the 2024 HPAIV H5 epizootic in dairy cows affecting 300 dairy herds in 14 US states. The cattle epizootic was spread mainly by milking machinery and animal transport. On affected farms infected cats developed fatal neurological diseases. Retail milk across the United States frequently contains viral RNA, but so far only a few milk farm workers have developed mild symptoms. The tracing of HPAIV with viral genome sequencing complicated the taxonomical naming of influenza viruses raising fundamental problems in how to mirror biological complexity in written plain language, rendering communication with the lay public difficult.

## Graphical abstract

With the help of intensive biological sampling and viral gene sequencing, the intrusion of high pathogenicity avian influenza viruses (HPAIV) into North America could be retraced. After reassortment with genes of local low pathogenic avian influenza viruses, HPAIV H5 caused in 2015 a major epizootic on poultry farms in the US Mid‐West and in 2024 an epizootic in dairy cows affecting 300 dairy herds in 14 US states. Figure credit: Hu et al. (2024).

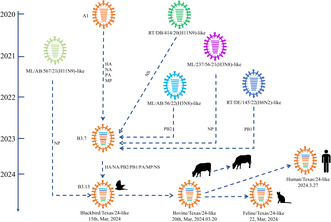

Under the pressures of the COVID‐19 pandemic, the scientific community, health authorities and the public at large asked which might be candidate viruses for a future pandemic. Influenza A viruses (IAV) clearly belong to this category as indicated by prior influenza pandemics. In addition, avian influenza viruses (AIV) are causing a panzootic in domesticated and wild birds as well as spill‐over infections in mammals, including infections in humans associated with high case fatality rates (Brüssow 2024). This Lilliput contribution reviews the arrival of high pathogenicity avian influenza viruses (HPAIV) into North America, where they caused two major epizootics (epidemics in animals), namely the large 2015 outbreak in poultry and then in 2024, infections in US dairy cows. The sequence of events leading to these two epizootics has been elucidated, but understanding its tracing needs some knowledge in influenza virus taxonomic classification (Figures 1 and 2). Before exploring the AIV taxonomy which might confuse and frustrate the casual reader, let us explore why the scientific language is necessarily complex and why scientific communication with a larger public represents a difficult task necessitating specific skills.

**FIGURE 1:**
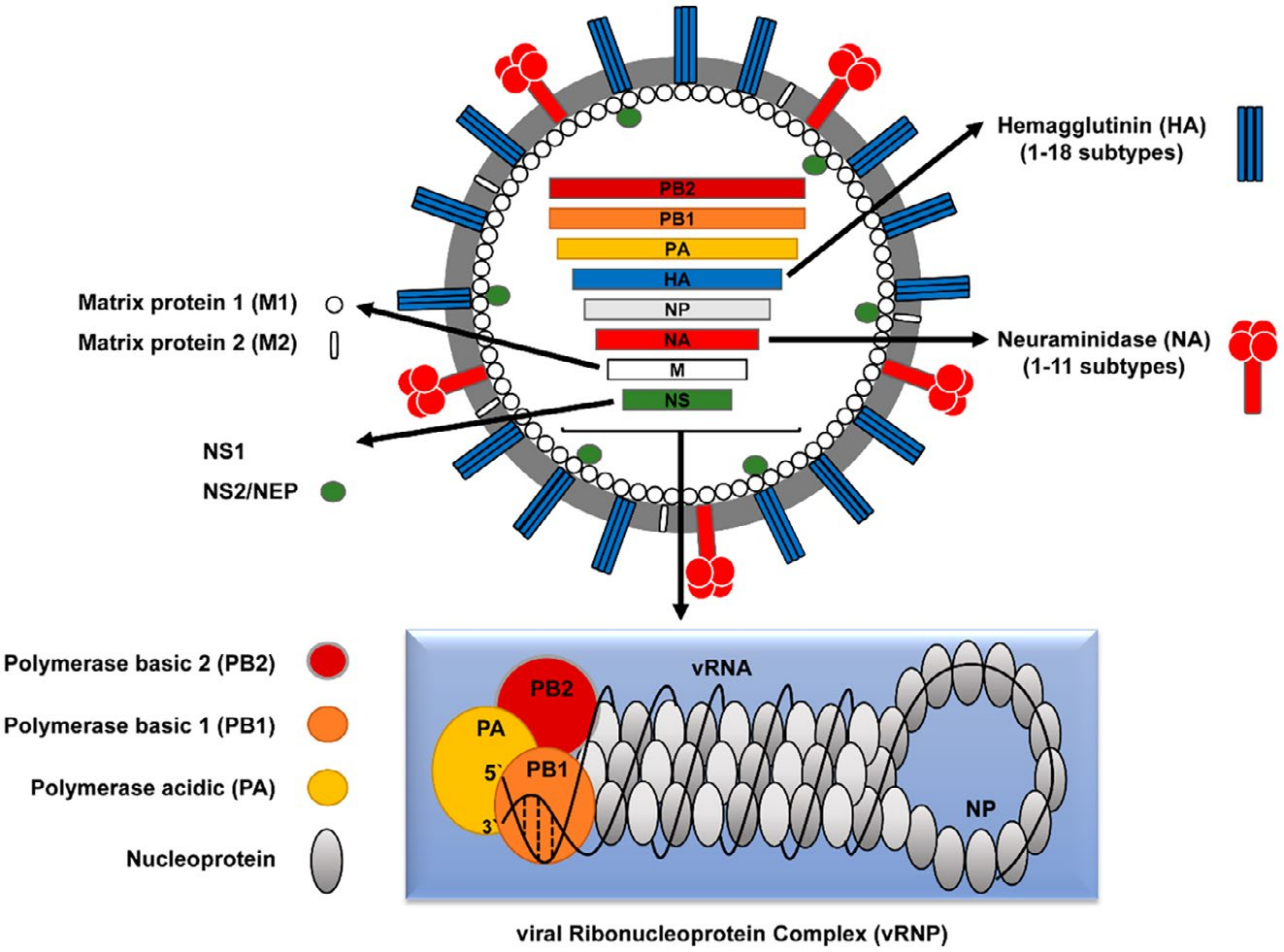
Schematic representation of the structure of an influenza A virus depicting the inside the viral genome consisting of eight single‐stranded separate RNA segments with their coding assignment for the RNA segments ordered in decreasing length. The arrows point from the gene segment coloured in the same way as the encoded protein symbol. The proteins are localised in the virion, and the insert below provides a more detailed representation of the viral RNA (vRNA) covered with the nucleoprotein and the three viral polymerase subunits. Figure credit: Mostafa et al. (2018).

**FIGURE 2:**
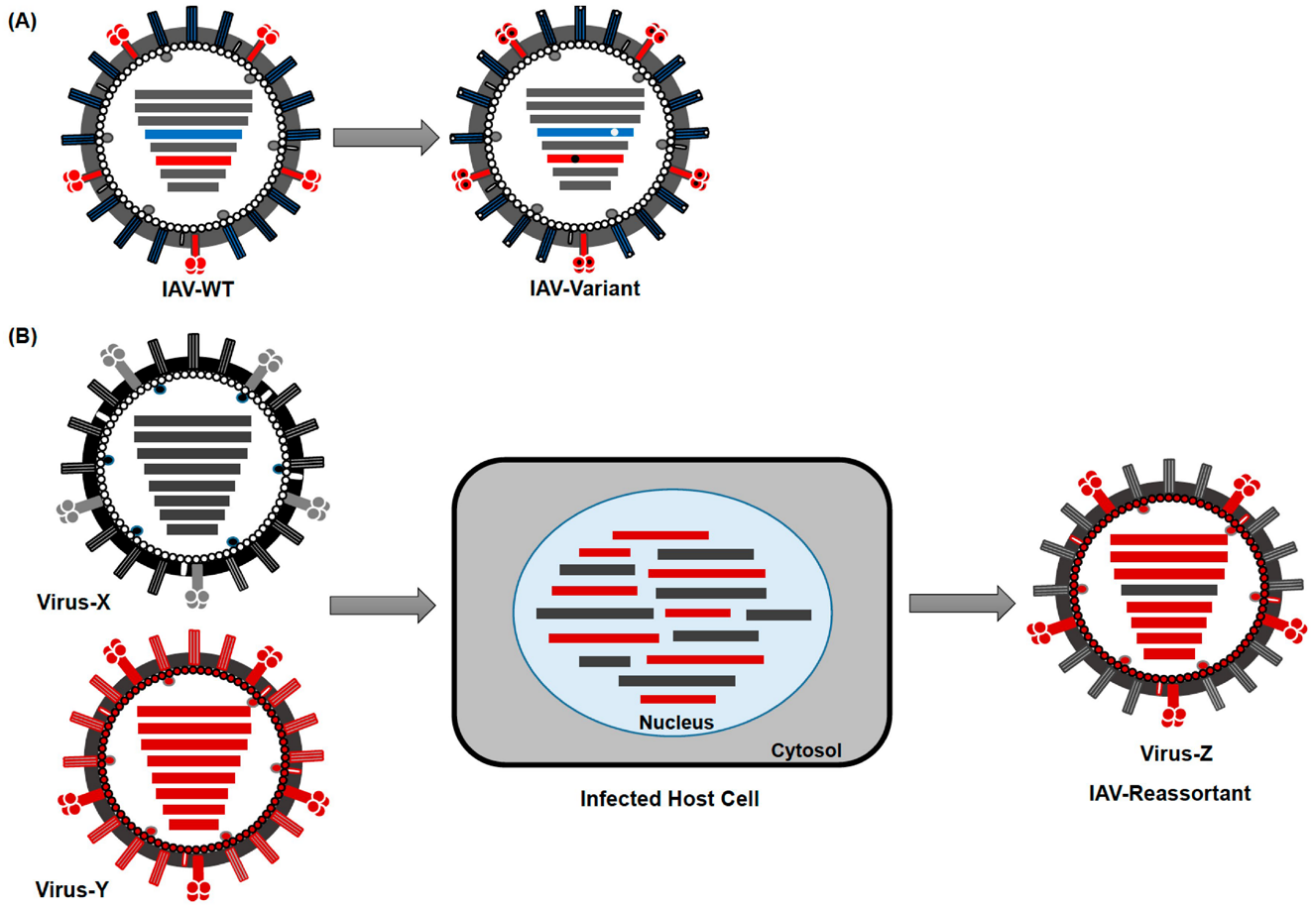
Influenza A viruses evolve by two distinct mechanisms. Viruses evolve by point mutations in the viral genes (depicted by small dots in the corresponding genes) leading to a variant virus by antigenic drift (A). Alternatively, when two different influenza A viruses happen to infect the same host cell, a reassortant virus might emerge that contains gene segments derived from either parent virus A or B by a process called antigenic shift. This process can create a vast variety of influenza A viruses that differ in genotype and phenotype since innumerable gene constellations are possible, only limited by the ecological fitness of the resulting reassortant viruses. For a concrete example, refer to Figure 3. Figure credit: Mostafa et al. (2018).

## 1 A Word on Scientific Naming

The lay audience frequently complains about the inaccessibility of the scientific language which represents a barrier for understanding scientific news and developments. If scientists would like their messages to be understood by a wider audience, they must find ways to present their discoveries using simpler wording. This is, however, a difficult task since it requires both scientific and communication skills. Basically, there is the following fundamental problem with scientific language and its understanding by laymen. Let us first consider the largest 20 volume Oxford Dictionary of English which comprises about 170,000 words. The English vocabulary is nearly twice as large as other Indo‐European languages since it derives its words from two language families. Then consider, for example, the number of animal species living on earth—the lower estimate is 3 million. Add to this the names of plants, fungi, bacteria and archaea and also the vocabulary needed to describe their life activities. One will easily end up with 20 million biological terms, and a comparable number of terms is needed by chemists to name chemical compounds and their reactions. We will for the purpose of argument arbitrarily neglect the terminology needs of other branches of knowledge. Obviously, the number of studied objects and phenomena will exceed the number of English words by several orders of magnitude. Then there is the problem of precise meaning of scientific terms which is not easily provided by the words of spoken languages. In science a name must be unambiguously linked with an object or phenomenon, preferably understood across several spoken languages. For the taxonomy of biological species, biologists have designed binomial Latin names. Some species names are easy, for example, the soil bacterium called *Bacillus subtilis* , while others are tongue twisters even for microbiologists, for example, the gut bacterium *Bacteroides thetaiotaomicron* . New words commonly based on Latin or Greek roots had to be created to classify the diversity of natural biological objects. Taxonomical committees supervise the name giving process and occasionally introduce (a sometimes routine‐disturbing) revision of genus names to account for new phylogenetic knowledge since biologists strive for a naming which reflects the natural evolutionary relationship of organisms. Name changes are proposed in specialised scientific journals (e.g., Zheng et al. 2020) before they are adopted by taxonomic committees.

That millions of scientific terms cannot be mirrored in 170,000 words of a given language is only one side of the communication problem. The other side is that few individuals (the greatest poets perhaps) master 100,000 words. Language skills are as a first approximation proportional to the years of school education. It was estimated that a well‐educated adult native speaker of English has a vocabulary of about 17,000 base words (Goulden, Nation, and Read 1990). Going from a productive to a receptive vocabulary will expand this number by two‐ or three‐fold. Less educated native speakers have a vocabulary of 3000 to 6000 words. Reading a daily newspaper needs at least 5000 words. Reading some prestigious scientific journals needs a much higher English vocabulary and text comprehension capacity, and that is an even greater problem because the majority of scientists are not English native speakers‐ communication problems exist therefore even between scientists.

**FIGURE 3:**
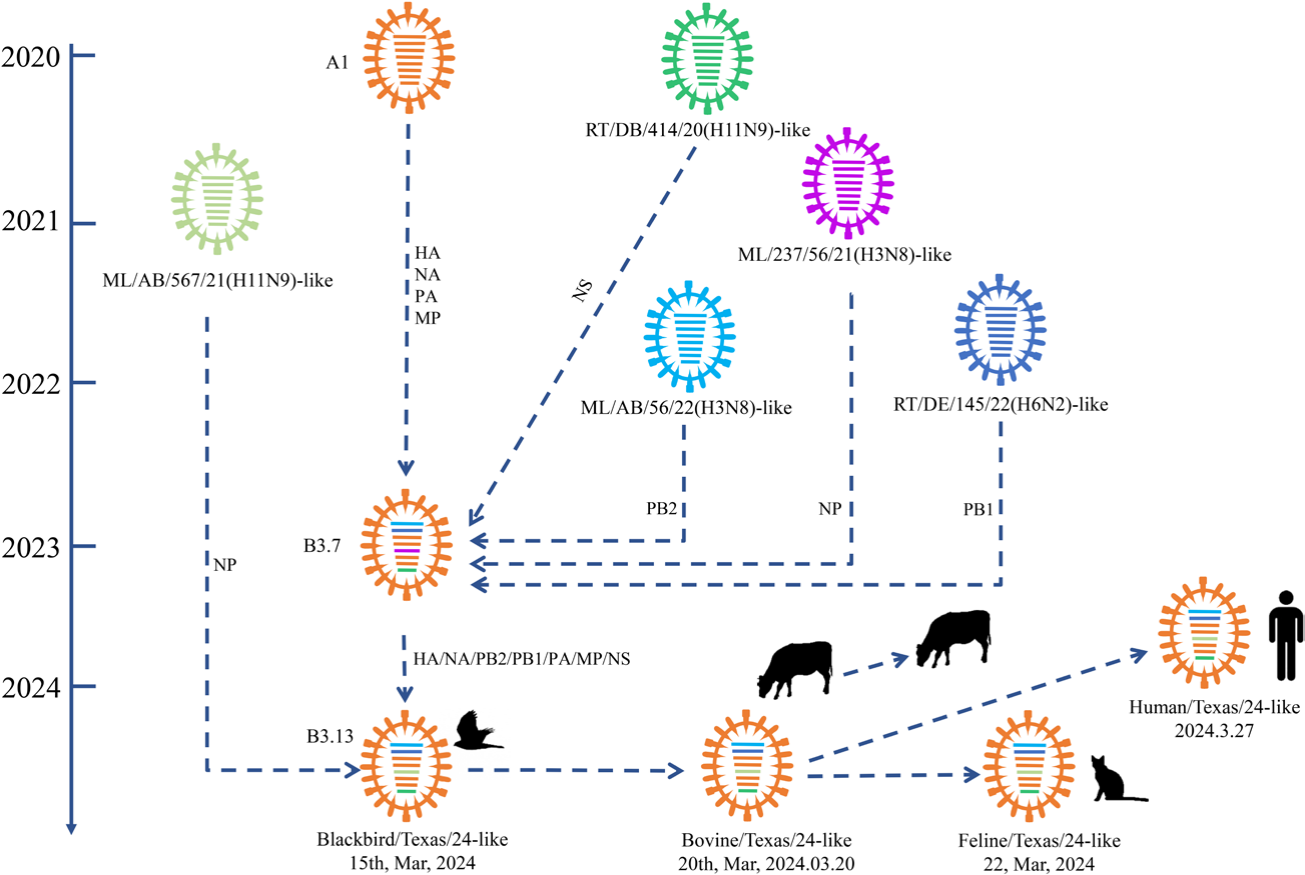
Schematic representation of genomic composition and reassortment time of HPAI H5N1 viruses from dairy cattle and other animals and humans in March 2024. Viral particles are represented by coloured ovals containing horizontal bars representing the eight gene segments (from top to bottom: PB2, PB1, PA, HA, NP, NA, M and NS). Each colour represents a separate virus background. The illustration is based on GenoFLU (https://github.com/USDA‐VS/GenoFLU) and phylogenetic analysis. AB, Alberta; DB, Delaware Bay; ML, Mallard; RT, Ruddy turnstone. Figure credit: Hu et al. (2024).

According to the general educational level of an audience and some knowledge of local school curricula, one can judge what scientific terms considered as basic can be used and what terms need a description or explanation in easier terms. Most scientists did not follow courses on scientific communication as part of their education at university. Communication is a tool that is not limited to the written word. The adage ‘A picture is worth a thousand words’ points to the importance of other ways of conveying information, ranging from the spoken word to visual displays. The point is illustrated in Figure 3 which retraces the evolution of the avian influenza virus causing the epizootic in US dairy cows. Explaining these events verbally would need a long and complicated text and the network of separate events would be difficult to explain while it can still be represented relatively clearly graphically. However, the graphical display is still complex, and the words of Oscar Wilde come to mind ‘The truth is rarely pure and never simple’. The visual abstract now requested by many scientific journals as a supplement to written abstracts is a tribute to our visual non‐verbal memory. Speaking might be a more effective way of communicating scientific knowledge than writing, particularly when a talk is supported by a live film. Scientists writing in scientific journals write for colleagues. When they want to reach a larger audience in popular science journals or social media such as podcasts or video conferences, they may want to collaborate with communication specialists or science journalists. Social media offer new possibilities for reaching even larger audiences, which might be especially important during a health crisis such as a pandemic where knowledge is initially limited and then acquired step by step. As informing the public is also a matter of trust, professional societies are perhaps better placed as a source of information rather than individual scientists. However, when targeting a wider audience, scientists should also consider non‐linguistic barriers to communication. A section of the public will have strong prejudices, particularly for biological issues (evolution, pandemics, vaccination, genetically modified organisms, climate change to name a few) which represent high hurdles for reaching these groups with arguments. The mere communication of scientific facts will not be sufficient to correct prejudices—a psychological, sociological and political knowledge of the deep roots of these prejudices and beliefs are needed to reach these people. Communication skills can be taught. In fact, in antiquity rhetoric was perhaps the most studied subject for young people starting a political career. One might be well advised to turn both to classical authors as well to marketing for learning how to formulate messages that you want to be understood and direct the actions of people. It would be a mistake to leave these techniques to demagogues for their selfish but frequently destructive goals. To stem mis‐ and disinformation one could also turn to AI technology as demonstrated in a controlled social trial with 2190 conspiracy believers engaged in dialogues with generative artificial intelligence. Interaction reduced significantly conspiratory beliefs, the reduction was durable and extended to unrelated conspiratory theories and had an impact on behavioural intentions (Costello, Pennycook, and Rand 2024).

## 2 The Complicated Taxonomy of AIV

Let us now return to naming problems in biology. Viruses are biological entities, but not organisms. If the application of the species concept is already contentious for prokaryotes, it does clearly not apply to viruses. The task of the International Committee on Taxonomy of Virus (ICTV) regulating the naming of viruses is thus not simple. The author has served on an ICTV subcommittee for bacterial viruses and a major problem was that these viruses do not evolve along linear lines of descent, but exchange gene modules between viruses (Brüssow 2009). A tree of life (already an illusion in bacteriology) thus quickly became an entangled net of relationships between bacterial viruses. Something similar but in even higher frequency occurs with influenza viruses which have a segmented genome (Figure 1) that can reassort into new gene constellations within the limits of their ecological fitness.

Due to the medical and veterinary importance of influenza viruses, this research field has developed its own terminology. Take for example an AIV which is at the root of the story reviewed in this Lilliput: the HPAIV isolate A/Goose/Guangdong/1/1996 (H5N1). This official name is unequivocal and informative: it refers to an individual virus from the family Orthomyxovirus belonging to the Alphagenus A (there are also B, C and D genera of influenza viruses) derived from a goose in Guangdong, China; more specifically, it was the 1st isolate of it made in 1996. A further classification of this specific virus isolate is given by its HN subtyping which refers to two viral surface proteins. H stands for the viral haemagglutinin HA protein, which is serologically differentiated into 16 subtypes (H1 to H16); N refers to the viral neuraminidase NA protein which is differentiated into 9 subtypes (N1 to N9) (neglecting here further H‐like and N‐like proteins). Isolate A/Goose/Guangdong/1/1996 (H5N1), shortened to Gs/GD, displays thus H5 and N1 proteins on its surface. However, influenza viruses consist of 8 single‐stranded RNA segments that upon double infection of a single cell with two influenza A viruses, can produce reassortant viruses where each segment can be derived from either one or the other parent virus only limited by the ecological competitiveness of the given gene constellation.

Veterinarians also use medically important phenotypic criteria to differentiate AIV. HPAIV causes high mortality in experimentally infected chickens, while low pathogenic viruses (LPAIV) result in mild disease or asymptomatic infection. A second distinction is that the HA protein of HPAIV displays a polybasic cleavage site which facilitates the activation of this viral surface protein by a larger number of cellular proteases to initiate infections (Figure 2).

When the Gs/GD virus caused in 1997 cross‐species infections in humans associated with deaths in Hong Kong, researchers started to monitor the movement of the virus from region to region. When following HPAIV, which were derived from the Gs/GD isolate through poultry epizootics and outbreaks in wild birds across Eurasia and North Africa, researchers noted that most original Gs/GD viral gene segments had undergone reassortment with influenza viruses circulating in birds leading to distinct genetic lineages. However, the haemagglutinin H5 protein gene from Gs/GD was not replaced since its emergence in 1996, underlining its importance for enhanced virulence and transmission. On the initiative of the World Health Organisation (WHO), the World Organisation for Animal Health or in French (it resides in Paris) Office International des Epizooties (OIE) and the Food and Agriculture Organisation (FAO), the H5N1 Evolution Working Group was created and designed an updated classification system for H5N1 HPAIV. Sponsorship by these three international organisations underlined already the medical, veterinary and agronomical importance of these influenza virus lineages. The working group proposed an extended classification based on the sequence analysis of the HA protein gene from H5N1 isolates which were circulating. In a H5 phylogenetic tree, the initial Gs/GD isolate defined a clade 0, from which clades 1 to 9 were differentiated. H5 genes from clade 2 showed a further marked splitting into subgroups, then called 2.1 to 2.5, where subgroup 2.3 had again to be split into further under‐subgroups, namely 2.3.1 to 2.3.4 (WHO/OIE/FAO H5N1 Evolution Working Group 2008). In a 2014 update, the phylogenetic tree analysis of nearly 8000 sequenced H5 genes led to further splits, necessitating a four‐digit branching scheme which was then further subdivided by the addition of letters. In the next sections we will investigate the spread of H5 HPAIV of clades 2.3.4.4 and 2.3.4.4b. We see here an interesting trend. The Latin binomial was replaced by a one line relatively plain text attribution which is still easily deciphered by a non‐specialised microbiologist. However, when it comes to the detective or ‘forensic’ work to retrace ‘culprits’ in the evolutionary march of this virulent virus through animals in epizootics, a finer‐grained taxonomy was needed. It turned out that for epidemiological purposes, the characteristics of only the H gene mattered, such that the taxonomy of HPAIV could be based on a single gene tree. Imagine the complex taxonomic denominator of AIVs if the veterinary important phenotype would have depended on the constellation of several distinct viral genes. Nature in biology has perhaps a ‘horror vacui’, in the sense that every possible ecological niche will be filled on earth, but Nature in biology has no horror of complexity. If needed, Nature would play (and probably has played) with countless combinations (here viral reassortants) to fill space through time while one might ask what degree of complexity can still be captured by the human mind.

Now let's return to AIVs. With the HN system supplemented by the alpha‐numerical H phylogenetic tree system, we are now in a position to trace the paths of the Gs/GD AIV descendants through epizootics. Some interesting patterns emerged: clade 1 AIV persisted since 2003 in the Mekong River Delta while clade 2.1 persisted in Indonesia. Clade 2.2.1.1 viruses are enzootic in Egypt while clade 2.2.2 is enzootic in Bangladesh (WHO/OIE/FAO H5N1 Evolution Working Group 2014). When the 2014 report was written, HPAIV H5N1 viruses from the Gs/GD lineage had not yet reached North America, which is surprising since the North American continent is linked with Asia via the Pacific flyway and with Europe via the Atlantic flyways of migratory birds. The apparent isolation of North America from Eurasia with respect to the spread of AIV is supported by two other observations. This viral barrier of unknown nature—an estimated 2 million migratory birds use the Pacific flyway—allowed that LPAIV could split into distinct lineages of Eurasian and North American viruses (Olsen et al. 2006). Furthermore, a survey of 500,000 wild birds sampled between 2006 and 2011 throughout the United States had not shown a single H5 AIV (Ip et al. 2015). For still unknown reasons, the situation changed in 2014 and dramatically so.

## 3 Towards the Greatest Poultry Epizootic in US History Introduced via the Pacific Flyway

In January 2014, an outbreak with an H5N8 HPAIV from the H5 Gs/GD lineage occurred in Korea, which was the first H5N8 epizootic observed outside China. The virus was attributed to the H5 clade 2.3.4.4. At this stage, three subgroups A to C (not to be mixed up with influenza A and C viruses), had differentiated within clade 2.3.4.4. Subgroup A and B AIV were isolated both on poultry farms and among wild birds near a lake wintering site in Korea. Subsequently, subgroup A AIV dominated the Korean epizootic. In September 2014 this virus was isolated from a long‐distance migratory bird in Eastern Siberia. Subgroup A viruses from Korean birds had apparently split in their Siberian grounds into three further subgroups called ic (intercontinental) A1 to 3. icA1 spread from Siberia to Europe and East Asia, icA3 to East Asia, and icA2 to East Asia and North America. icA2 was detected in November and December 2014 among multiple bird species along the west coast of North America (Lee et al. 2015, 2017, 2018).

This detective work of viral spread was only possible because a worldwide sampling of influenza viruses from humans and animals is routinely done with high frequency and wide geographical coverage. Sequencing and phylogenetic analysis without this laborious groundwork would not have traced these and other infection events described below. Such dedicated field work also points the way for pandemic preparedness efforts to be developed for other viruses representing pandemic threats.

In late November 2014 an outbreak with 70% mortality was noted in British Columbia/Canada on a large meat turkey farm. A nearby broiler breeder flock experienced at the same time severe pulmonary congestion with a 10% mortality rate. Sequence analysis identified an HPAI H5N2 virus, the first HPAI outbreak in North America that involves a virus with a Eurasian Gs/GD‐lineage HA gene. In fact it was a reassortant virus which derived 5 gene segments (PB2, PA, HA from Eurasian clade H5 2.3.4.4 lineage, M and NS from Eurasian HPAI H5N8) and the remaining gene segments (PB1, NP and NA) from North American lineage waterfowl viruses (Pasick et al. 2015). Alerted by this viral intrusion, USDA conducted surveys and detected H5N8 viruses of the H5 2.3.4.4 lineage in two wild ducks at 30 km distance from the outbreak location. Falcons fed on the infected ducks died from infection (Ip et al. 2015). In February 2015 an outbreak with a HPAIV H5N1 belonging to clade 2.3.4.4 was observed in backyard chickens from British Columbia. Sequence analyses demonstrated that PB2, HA, NP and M were derived from a Eurasian lineage H5N8 virus and the remainder of the genes from North American LPAIV lineages. The isolate was very lethal for turkeys in experimental infections, and moderately pathogenic in geese, but caused only mild signs in ducks despite comparable viral shedding. Pancreatic and hepatic necrosis was observed. Low doses of the isolate were highly virulent in mice while no clinical signs or viral shedding were detected in experimentally infected pigs (Berhane et al. 2016). Phylogenetic network analysis constructed from the HA‐encoding gene suggested that these viruses travelled with migratory birds that had their breeding grounds in eastern‐most Siberia crossing Beringia and followed the Pacific flyway along the western coast of North America used by about 2 million aquatic migratory birds (Lee et al. 2015).

In January 2015 the US Department of Agriculture (USDA) conducted an HPAIV survey in 4700 opportunistically collected birds harvested by hunters along the entire Pacific coast of the United States. Ten per cent of the birds tested positive for AIV, and 1.3% were positive for the H5 2.3.4.4 lineage (with the following frequency: H5N2 = H5N8 > H5N1). Two duck species, *Anas americana* and *A. platyrhinchos* (mallard), showed the highest frequency of HPAIV. Many waterfowl species were susceptible to infection but did not display signs of clinical disease, rendering future control measures difficult. Between December 2014 and February 2015 another survey of 150 sick or dead birds revealed that 6% tested positive for H5 2.3.4.4 icA (Bevins et al. 2016).

In April 2015, HPAIV H5N2 case numbers increased markedly on commercial poultry farms in the Midwest US and reached a peak at the end of April 2015. Overall, 211 commercial premises were affected in 9 Midwest US states. A total of 43 million chickens (primarily layers representing 10% of the US inventory), and 7.4 million turkeys (representing 7% of the US inventory) died from the disease or were destroyed to contain the epizootic. Infection signs included decreased food and water consumption, coughing, sneezing, decreased or deformed egg production, sudden death, swelling and purple discoloration of the animals, loss of coordination and diarrhoea. Sharing equipment between farms, entry of wild birds into barns and travelling farm workers were contributing to the virus spread. HPAIV could be aerosolized from infected flocks and remain airborne. By imposing rigorous control measures, the last case was observed in mid‐June 2015. The economic consequences of this outbreak were substantial: $1.6 billion in direct losses from turkeys and chicken; another $1.6 billion for restocking the farms; a doubling of consumer prices for eggs; and trade bans suffered for eggs and poultry. USDA qualified this outbreak as perhaps the most significant animal health event in US history. Overall, 3400 people were deployed for the outbreak control, yet no human infections were observed. In parallel, no mutations known to be associated with increased transmission of AIV to mammals were detected (USDA 2016a).

Phylogenetic analysis of the outbreak strains revealed two major groups, both were found in wild birds travelling along the Pacific and Mississippi flyways. Multiple independent HPAIV H5N2 introductions with viruses of wild bird origin were deduced, but case amplification was largely caused by extensive between‐farm virus transmission (Lee et al. 2018).

Biosecurity measures, quarantine and targeted culling led to the end of the US poultry epizootic in mid‐June 2015. After this poultry outbreak, 6700 migratory ducks were sampled mainly from the Mississippi flyway; they yielded 370 AIV, but surprisingly no HPAIV H5Nx isolates. Apparently, these high pathogenicity AIV failed to get established in North America's wildfowl. The scientists discussed increased bird population immunity and lesser fitness of these isolates in wildfowl as reasons for the lack of their persistence in the environment (Krauss et al. 2016). Indeed, when USDA sampled 40,000 wild birds in the year following the 2015 poultry outbreak from all flyways of migratory birds in the United States (Pacific, Central, Mississippi and Atlantic flyways), no HPAIV cases were confirmed (USDA 2016b). These observations contrast well with the situation before the poultry outbreak when HPAIV H5 isolates were a frequent finding in wildfowl (Krauss et al. 2016). However, there is no clear evidence that the disappearance of HPAIV H5Nx isolates from North America after the 2015 outbreak was due to the build‐up of immunity in the wild bird population (Ramey et al. 2018).

## 4 A Second Intrusion of H5 HPAIV Into North America via the Atlantic Flyway

HPAIV returned to North America in December 2021. On an exhibition farm in Newfoundland, Canada, a die‐off was observed, killing three‐fourths of the birds. Symptoms in chicken and turkeys included swollen heads and cutaneous haemorrhage. Wild birds had intermingled with the captive birds on the farm. Sequencing demonstrated that all eight gene segments of the Newfoundland AIV isolated from this outbreak had a Eurasian origin and were derived from the Gs/GD isolate. Researchers attributed the infecting strain to clade 2.3.4.4b H5N1 viruses circulating in Europe since spring 2021 without any genetic admixture from local North American AIV. Phylogenetic tree analysis indicated that the ancestor of the Newfoundland isolate was circulating in Northwest Europe during the 2020/2021 outbreak in Europe. The most likely Atlantic flyway used by the bird viral vectors was one with a stop in Iceland, which is used annually by more than 2 million birds. Researchers suggested the Eurasian wigeon, a dabbling duck, as the most likely virus carrier because wigeons are frequently detected as the first species to be HPAIV positive without displaying clinical signs (Caliendo et al. 2022). The Iceland flyway was confirmed by the sequencing of HPAIV H5N1 isolates from gannets, gulls and geese found dead in Iceland which could be linked to the Newfoundland virus. The sequence data pointed to a UK origin of these clade 2.3.4.4b viruses in Iceland (Günther et al. 2022).

The newly introduced clade 2.3.4.4b viruses then spread through North America. Wild bird collection routinely conducted by USDA revealed HPAIV H5N1 isolates closely related to the Newfoundland isolates along the US Atlantic coast (particularly North and South Carolina) implicating again dabbling ducks as likely virus carriers. Their migration range covers the Eastern half of the continental US (Bevins et al. 2022). Veterinarians noted in 2022 two waves of excess mortality among birds in Maine. The Spring wave affected raptors, the summer wave killed gulls and eider. During the summer waterbird infection wave, an increased number of stranded dead seals were observed, and also a quarter of seals tested positive for the clade 2.3.4.4b HPAIV which circulated at the same time in the water birds. Seals showed viral titres in nasal, oral, conjunctival and rectal swabs (Puryear et al. 2023). Clade 2.3.4.4b viruses closely related to the Newfoundland avian isolates without admixture of LPAIV genes were also detected in three black bears (a mother and two cubs) from Quebec, Canada, in June 2022. Viral antigens were found in the liver and the brain of the bears and were associated with severe necrosis (Jakobek et al. 2023).

Opportunistic samples collected between April and July 2022 by citizens from diseased or dead wild mammals also revealed clade 2.3.4.4b viruses that contained at least two gene segments (PB1, NP) from North American LPAIV. The infected animals were distributed across ten US states stretching from the East to the West coast and included mainly red foxes, but also skunks, racoons, bobcats and opossums, indicating a wide host range of 2.3.4.4b viruses among wild mammals. Brain lesions were prominent which corresponded to the observed neurological clinical signs. Most of the isolated HPAIV showed no genetic adaptation for infection of mammals (Elsmo et al. 2023).

Now permit a further dose of genetic differentiation which allowed the tracing of epizootics, but at the same time further complicated the AIV taxonomy. Between December 2021 and April 2022, 13,000 wild birds and 500,000 domesticated poultry samples were tested for HPAIV across the United States. Three independent H5N1 clade 2.3.4.4b virus introductions were detected, two spread westward with migrating wild birds from New England states (called A1, A2) and one originated in Alaska (called A3). While spreading, up to five internal gene segments were introduced from LPAIV into A1, resulting in reassortants called B1 to B5. For example, B3 goes back to a reassortant event occurring in North Dakota in January 2022 with a mallard LPAIV from where this virus spread to neighbouring states around the Great Lakes. According to the number and combination of reassorted LPAIV gene segments appearing in clade 2.3.4.4b viruses, subtypes of B3 were differentiated, numbered as B3.1, B3.2 and so on. Most detections of A1 and its derivatives in domestic birds were shown to be point‐source transmissions from wild birds, with limited farm‐to‐farm spread (Youk et al. 2023).

In 2022 another group of researchers isolated HPAIV clade 2.3.4.4b viruses from wild eagles, hawks and diving ducks. All isolates represented reassortant viruses containing two to four gene segments (PB1, PB2, PA, NP) from North American LPAIV lineages. When tested in the ferret mammalian infection model, increased virulence was associated with increasing numbers of North American gene segments. Higher virulence in ferrets correlated with higher viral titres and wider tissue distribution of the virus. The observations were reproduced in a mouse infection model (Kandeil et al. 2023). In contrast, HPAIV H5N8 and H5N2 viruses of the 2.3.4.4 lineage isolated in North American during the 2014/2015 outbreak showed only low viral titres and minimal clinical signs in experimentally infected mice and ferrets (Kaplan et al. 2016) consistent with the lack of reports of spillover infections into mammals during the 2015 poultry outbreak in the United States.

## 5 H5 Epizootic on US Dairy Farms

In February 2024, Texan farmers observed in dairy cows reductions in milk production, decreased feed intake and thickened yellow milk resembling colostrum. Laboratory investigation identified influenza A virus in milk. The clinical syndrome affected mainly older cows in mid to late lactation and subsided within 2 weeks after onset. By early March similar cases were seen in milking cows from Kansas and New Mexico. At the time of writing (October 2024) the US Centers for Disease Control and Prevention (CDC) reported 296 affected herds in 14 US states. California led with 96 infected herds, followed by Colorado, Idaho, Michigan and Texas. Sequencing confirmed H5N1 HPAIV from clade 2.3.4.4b subtype B3.13 (Caserta et al. 2024). B3.13 is a reassortant between B3.7 virus and an LPAIV from North America contributing one gene segment coding for NP (Hu et al. 2024). Figure 3 reproduces a graphical display of the origins of the HPAIV causing the 2024 epizootic in cattle depicting the origin of its gene segments and the sequence of events leading to the epizootic viral strain (quoted from Hu et al. 2024). The HA gene segment from viruses of the dairy outbreak formed a single monophyletic clade, suggesting a recent single spillover event from wild birds. The spillover was dated back to November/ December 2023, followed by several months of silent transmission on farms and further interstate transmission by cattle movement according to current production practices (bioRxiv preprint doi: https://doi.org/10.1101/2024.05.01.591751). To stem further spread of infection, cattle have now to be tested negative for H5N1 to qualify for interstate transport in the United States.

Symptoms in HPAIV‐infected milking cows included in addition to the above‐reported signs lethargy and dehydration, while respiratory symptoms were only mild. The drop in milk production occurred suddenly and ranged from 20% to 100% and continued at least 2 weeks beyond the resolution of symptomatic illness. Between 3% and 20% of animals on affected farms became infected. Infected cows seroconverted but viral replication was largely restricted to the mammary gland where mastitis developed. In infected cows, only low viral titres were seen in nasal secretions or urine and none in faeces. In contrast, viral titres were high in milk reaching up to 10^**9**^ infectious viruses per ml. Milk viral RNA load decreased gradually over a month, but infectious virus was not detected after 2 weeks following symptom onset. Sequence analysis of the isolated viruses suggested short‐range transmission between nearby farms possibly mediated by infected blackbirds. Long‐distance transmission between Texas and Ohio probably occurred via interstate transport of sub‐clinically infected milking cows (Caserta et al. 2024).

Deaths of wild birds and domestic cats were observed on affected dairy farms. Two studied cats showed depressed mental state, stiff body movements, ataxia and blindness. Pathological examination revealed a systemic virus infection associated with severe meningoencephalitis. HPAIV was detected in the brain, lung, heart and retina of the cats. Viral titres were very high in the brain, consistent with the severe neurological symptoms. The virus from cats was nearly identical to that found in the milk of infected cows suggesting transmission of the infection via unpasteurized milk from infected cows fed to cats (Burrough et al. 2024). Also, wild birds (blackbird, grackle), poultry and a raccoon found near the affected farm were infected with a virus that nested within the cattle virus clade suggesting viral spillover (or spillback) from cows to birds and other mammals.

Subsequently, it was tested in experimental infections whether the virus from the milk of naturally HPAIV‐infected cows could induce disease in orally exposed mice (to mimic food‐borne transmission). This was the case: mice challenged with 10 μL milk, but not those challenged with lower amounts showed weight loss and 60% mortality. High viral titres were detected in the nose, lung and brain of mice. Upon intranasal inoculation (to mimic respiratory transmission) of mice, a low lethal dose (LD_50_) of 32 plaque‐forming units (PFU) was determined for mice with the HPAIV from cow's milk. All mice challenged with 10^**3**^ PFU succumbed to infection. Intranasal inoculation of mice caused a systemic infection affecting many organs: high viral titres were seen in the mammary gland, brain, trachea and lung. Transfer of the infection from infected lactating mice to their suckling offspring occurred in some, but not all maternal‐offspring pairs. Ferrets infected with HPAIV from cow's milk failed, however, to transmit the infection via the respiratory route to ferrets in adjacent cages. In vitro HPAIV bound to both the avian virus‐specific α2,3‐ and human virus‐specific α2,6‐linked sialic acids, raising the possibility that these viruses might also infect cells of the upper respiratory tract of humans. This property might have been acquired during passage of HPAIV in cows (Eisfeld et al. 2024) since all US H5N1 isolates from 2022 showed the sialic acid receptor binding specificity of avian and not that of mammalian influenza viruses (Kandeil et al. 2023).

In a German‐US collaboration, six calves were oro‐nasally inoculated with 10^**6**^ B3.13 virus isolated from a cow in Texas. Only occasional and very mild clinical symptoms were observed. In contrast, when six lactating cows were inoculated by intramammary instillation with the same dose of the Texan cow virus or a European H5N1 wild bird isolate, severe clinical disease was observed that led in 3 cows to unscheduled early euthanasia. Clinical signs included postural abnormalities, lethargy and high fever. Reduced feed intake and greatly reduced milk production were noted 2 days after inoculation and persisted for a further 3 weeks. Viral shedding was minimal to negative when investigating many clinical samples, except for high viral titres in milk in the week after inoculation. Viral titres dropped when antiviral antibodies appeared in milk 10 days post infection. Pathological examination of 40 tissues 3 weeks after inoculation (the end of the experiment) showed that in calves, virus replication was limited to mucosa‐associated lymphoid tissue. In cows high viral replication levels were detected in the mammary gland, low titres were seen in neural tissue and none in the respiratory tract. Histology demonstrated severe mastitis. Three sentinel calves co‐mingled with the inoculated calves remained uninfected. A possible adaptive mutation PB2 E627K emerged after intramammary replication (Halwe et al. 2024). That virus replicated in the udder, but not in the respiratory tract parallels the expression of the avian α‐2,3 sialic acid receptor in the udder, but not in the upper respiratory tract of cattle (Kristensen et al. 2024). A study from USDA reported similar outcomes when challenging 4 heifers and 2 milking cows with a Texan B3.13 virus by an aerosol respiratory route and an intramammary route, respectively. Heifers showed low‐level viral replication in the epithelia lining of the bronchi. The researchers observed low‐frequency variants associated with mammalian adaptation in two viral proteins, NS (F103S) and PB2 (L631P) (bioRxiv preprint doi: https://doi.org/10.1101/2024.07.12.603337).

Sporadic infections with HPAIV H5N1 linked with up to 50% mortality have been observed in many Eurasian countries over the last 20 years (Brüssow 2024). Scientists and clinicians were therefore looking for possible human cross‐infections with the ‘bovine’ B3.13 virus during the 2024 US outbreak. Cases were indeed identified. In March 2024, a US dairy farm worker from Texas developed mild conjunctivitis. A conjunctival swab specimen showed a high‐tittered B3.13 virus related to that from affected milking cows. The conjunctivitis resolved spontaneously, and no contact persons were infected (Uyeki et al. 2024). In May 2024 two dairy farm workers from Michigan were infected with H5N1 AIV. One person developed mild self‐resolving conjunctivitis after suffering milk splashes into his eye. The conjunctival swab contained the H5N1 virus, but not the nasopharyngeal swabs. The other farm worker, caring for an ill cow, developed one day after exposure a cough, shortness of breath, sore throat and fatigue despite protecting himself with gloves, eye protection, but no mask. The illness resolved itself within a week (Morse et al. 2024). In July 2024, the CDC reported a fourth infected farm worker who showed eye symptoms that resolved after oseltamivir treatment (CDC Reports Fourth Human Case of H5 Bird Flu Tied to Dairy Cow Outbreak | CDC Online Newsroom | CDC). By mid‐October 2024, the CDC added six further cases in dairy farm workers from California and nine cases in poultry farm workers from Colorado (H5 Bird Flu: Current Situation | Bird Flu | CDC). Human cross‐infections occurred, but symptoms remained mild and no cases of person‐to‐person transmission were seen.

Since B3.13 infected cows shed high numbers of B3.13 viruses into milk, the question arose to what extent commercial milk is contaminated and whether standard pasteurisation kills viral infectivity. Indeed, molecular tests detected H5N1 viral RNA in 20% of pasteurised retail dairy products from over the United States in 2024 with mean titres of 10^3^ virus per g dairy product and maximal titres of 10^5^ per g. No virus was detected in yogurt samples (Spackman et al. 2024). Other groups detected that 36% of convenience samples of retail dairy products showed molecular evidence of H5N1 clade 2.3.4.4b RNA. However, no infectious virus was observed when tested in cell culture, eggs or mice. The researchers recommend commercial milk tests to assess the spread of the bovine epizootic (Detection of A(H5N1) influenza virus nucleic acid in retail pasteurized milk (researchsquare.com)) as an addition to wastewater surveillance (Louis et al. 2024). In further tests, untreated milk samples from an affected herd in New Mexico showing 10^5^ to 10^7^ infectious virus were fed to mice. Mice showed lethargy and high viral titres in the respiratory organs. Five minutes of heat treatment at 63°C reduced viral infectivity for cell culture by 5‐logs and pushed the virus below the detection threshold. Heat treatment at 72°C for up to 20 s still allowed some infectivity survival when tested in eggs as a sensitive detection system (Guan et al. 2024). In another series of experiments 10^6^ virus from a mammalian carnivore host was diluted into raw cow's milk. Heating at 63°C for 2 min eliminated viral infectivity for cell culture. At 72°C, viral infectivity decreased by 4 logs within 5 s. However, the infectious virus remained just above the assay detection limit after 20 s treatment at 72°C (Kaiser et al. 2024).

## 6 Outlook

Based on currently available information, FAO‐WHO‐OIE assesses the global public health risk of HPAIV (H5N1) viruses as low (joint‐fao‐oie‐who‐preliminary‐risk‐assessment‐associated‐with‐avian‐influenza‐a(h5n1)‐virus.pdf). This evaluation pertains to the infection risk for humans. However, for wild and domesticated birds HPAIV H5Nx is already a major panzootic. Another concerning aspect is the wide host range of these HPAIV, covering not only many avian species but also numerous mammalian species which challenges the concept of species barriers for viral infections. The concept relies on the notion that virus infections—contrary to most bacterial infections—rely on an obligatory intracellular replication of the viral pathogen in the host cell. Many host gene functions are needed to achieve a successful viral replication and the genetic differences between different host species make it hard for a virus to be promiscuous with respect to infecting different hosts. The viral species barrier has never been a clear rule, but the ease with which HPAIV infects species belonging to different vertebrate classes is a source of concern. Replication is not the only constraint for viral infections to take hold, a virus must also achieve efficient transmission. HPAIV has demonstrated not only in the 2015 epizootic on US poultry farms that it has achieved this for domesticated avian host species. The efficient transmission of HPAIV among milking cows from the United States demonstrates that also for this part of viral propagation, HPAIV has found a solution in mammals. Dairy cows are not the only cases: HPAIV H5N1 has achieved efficient transmission in Europe on fur farms (mink and foxes in Spain and Finland); long‐range transmission of H5N1 was seen in South American marine mammals (reviewed in Peacock et al. 2024). With respect to farmed animals, one might argue that agricultural practices have helped viral transmission. In the US dairy cow HPAIV outbreak, the viral spread has certainly been assisted by the use of milking machinery and the interstate transport of infected cattle. However, the epizootics in sea mammals demonstrate that HPAIV managed transmission also among mammals under natural conditions, which poses threats to wildlife already threatened by environmental change.

Rating epizootics as a public health concern only with respect to the occurrence of human cross‐infections might be short‐sighted. If epizootics like that in US poultry in 2015 and that in US dairy cows in 2024 would run out of control and expand, it could have serious cost consequences for food supply in societies relying strongly on animal products in the form of eggs, milk and meat. In traditional pastoral societies relying on herding the consequences can be dramatic. The rinderpest epizootic in Africa from 1890 killed in excess of 5 million cattle. Without infecting humans, the epizootic killed an estimated third of the human population in Ethiopia and two‐thirds of Maasai in Tanzania by starvation via destroying the economic basis of their lifestyle (Normile 2008). Dairy cows recover from H5N1 infection spontaneously, and human starvation in affluent societies is thus an unlikely spectre. However, the public should realise the possible impact of emerging viral infections in domesticated food animals and fungal infections in plants. The One Health concept, seeing human, animal and environmental health in a single context, is thus an important new framework for looking at public health. HPAIV raises also concerns for causing severe neurological diseases in cats. In the United States there are about 30 pet cats per 100 people (350 million pet cats are estimated worldwide). In view of the strong emotional links of cat owners to their cats, the psychological impact of a pet cat epizootic with HPAIV should not be underestimated.

The HPAIV epizootics in North America have also important learning lessons for pandemic preparedness. Retracing these epizootics was only possible by intensive sample collection and sequencing. These efforts are very labour intensive and cannot be achieved by professionals alone, sampling also needs the participation of citizen scientists. Bird watching is very popular in some countries and amateurs could be motivated to report unusually high numbers of diseased and dead birds or animals in general. Campaigns to recruit citizen scientists could also heighten awareness for the One Health concerns in a larger public and openness to the scientific method in societies that are increasingly influenced by mis‐ and disinformation in social media, not the least on pandemics. Citizen scientists could thus not only be helpful for data collection and sampling but could also serve as messengers for responsible citizens and help rationalise the public discussion.

Pandemic preparedness in the case of HPAIV also requires other practical considerations. Seasonal influenza virus human vaccines do not protect against HPAIV. H5‐specific flu vaccines can be produced by current technologies, and one might consider producing and stockpiling such vaccines, at least for health professionals, should human‐to‐human transmission of HPAIV become one day an issue. Current antivirals are efficient against HPAIV, but this might not remain so. Funding efforts against possible pandemics will be difficult in times of stressed public health budgets. Raising attention to these problems in the public might therefore be an important preparatory step and that leads back to the initial problem of scientific communication, namely the problem to mirror complex scientific issues in an understandable language, a difficult but increasingly important challenge for the scientific community. The increasing complexity of taxonomical naming of HPAIV illustrates not only the difficulty of scientific communication with the lay public but also the flexibility of Influenza viruses via reassorting gene segments from HPAIV and LPAIV isolates from double infections in a single host. The intensive sampling and sequencing of AIV allowed not only to reconstruct and trace viral lineages through time and space but demonstrated also the high dynamic of a segmented viral genome. So far, the reassortants described in this review are those between HPAIV and LPAIV. The spillover of these reassortants into mammalian species raises the concern for the emergence of avian‐mammalian reassortant influenza A viruses. In dairy cows, this risk is low because so far only a few influenza D viruses have been reported for cattle and no reassortment has so far been observed between Influenza A on one side and influenza B, C and D viruses on the other side. However, marine mammals are frequently infected by Influenza A viruses potentially allowing avian‐mammalian reassortants to emerge, which might then represent more dangerous public health threats.

## Author Contributions

**Harald Brüssow:** conceptualization (lead), writing – original draft (lead).

## Conflicts of Interest

The author declares no conflicts of interest.

## Data Availability

Data sharing not applicable to this article as no datasets were generated or analysed during the current study.
